# *Neisseria gonorrhoeae* Modulates Immunity by Polarizing Human Macrophages to a M2 Profile

**DOI:** 10.1371/journal.pone.0130713

**Published:** 2015-06-30

**Authors:** María Carolina Ortiz, Claudia Lefimil, Paula I. Rodas, Rolando Vernal, Mercedes Lopez, Claudio Acuña-Castillo, Mónica Imarai, Alejandro Escobar

**Affiliations:** 1 Instituto de Investigación en Ciencias Odontológicas, Facultad de Odontología. Universidad de Chile, Santiago, Chile; 2 Center for Integrative Medicine and Innovative Science, Facultad de Medicina. Universidad Andrés Bello, Santiago, Chile; 3 Departamento de Odontología Conservadora, Facultad de Odontología. Universidad de Chile, Santiago, Chile; 4 Programa Disciplinario de Inmunología, Instituto de Ciencias Biomédicas, Facultad de Medicina. Universidad de Chile, Santiago, Chile; 5 Laboratorio de Inmunología, Departamento de Biología, Facultad de Química y Biología. Universidad de Santiago de Chile, Santiago, Chile; University of Michigan Health System, UNITED STATES

## Abstract

Current data suggest that *Neisseria gonorrhoeae* is able to suppress the protective immune response at different levels, such as B and T lymphocytes and antigen-presenting cells. The present report is focused on gonococcus evasion mechanism on macrophages (MФ) and its impact in the subsequent immune response. In response to various signals MФ may undergo classical-M1 (M1-MФ) or alternative-M2 (M2-MФ) activation. Until now there are no reports of the gonococcus effects on human MФ polarization. We assessed the phagocytic ability of monocyte-derived MФ (MDM) upon gonococcal infection by immunofluorescence and gentamicin protection experiments. Then, we evaluated cytokine profile and M1/M2 specific-surface markers on MФ challenged with *N*. *gonorrhoeae* and their proliferative effect on T cells. Our findings lead us to suggest *N*. *gonorrhoeae* stimulates a M2-MФ phenotype in which some of the M2b and none of the M1-MФ-associated markers are induced. Interestingly, *N*. *gonorrhoeae* exposure leads to upregulation of a Programmed Death Ligand 1 (PD-L1), widely known as an immunosuppressive molecule. Moreover, functional results showed that *N*. *gonorrhoeae*-treated MФ are unable to induce proliferation of human T-cells, suggesting a more likely regulatory phenotype. Taken together, our data show that *N*. *gonorroheae* interferes with MФ polarization. This study has important implications for understanding the mechanisms of clearance versus long-term persistence of *N*. *gonorroheae* infection and might be applicable for the development of new therapeutic strategies.

## Introduction

*Neisseria gonorrhoeae* is the etiological agent of the sexually transmitted disease gonorrhea, with a worldwide incidence and an estimate of over 100 million new infections per year [1]. In women, infection by *N*. *gonorrhoeae* is associated with several clinical manifestations such as urethritis, cervicitis, pelvic inflammatory disease, ectopic pregnancy, chronic pelvic pain and infertility [2]. Moreover, gonococcus (GC) is often co-morbid with other STDs such as HIV, which increases the risk of transmission of this disease [3, 4]. Due to its increasing antimicrobial resistance and the absence of effective vaccines [5], gonorrhea remains as an important public health issue.

The gonococcal infection is unable to induce a state of protective immunity. This is supported by clinical data indicating that previous infections with *N*. *gonorrhoeae* do not improve the immune response and gonorrhea can be repeatedly acquired [6, 7]. The mechanisms of immune evasion exhibited by the pathogen are multiple and involve the innate and adaptive immune response [8–11]. Studies in the murine model of gonococcal genital tract infection show an increase of CD4+Foxp3+CD25+ regulatory T lymphocytes (Tregs) in the lymph nodes draining of the genital tract. This increase correlates with an augmentation of transforming growth factor (TGF)-β positive cells in the uterine stroma of infected animals [8]. In addition, *N*. *gonorrhoeae* enhances TGF-β production and thereby promotes a Th17-dependent response with the concomitant suppression of Th1/Th2 protective responses [9]. Recently, Liu *et al* demonstrated that Th1/Th2 responses are suppressed by mechanisms dependent on TGF-β and interleukin (IL)-10 as well as type 1 regulatory T (Tr1) cells [10]. Moreover, the interaction of gonococcal pili with CD4+T cells induces the activation and proliferation of lymphocytes and stimulates the secretion of IL-10 [11]. In contrast, Opa proteins mediate the binding to CEACAM-1 expressed by CD4+ T cells and suppress activation and proliferation of naive lymphocytes [12, 13].

Macrophages (MΦ) and dendritic cells (DCs) are critical cells in the innate immune response, acting as sentinels in peripheral tissues and responding against pathogens sensed in the environment. In this regard, it has been showed that *N*. *gonorrhoeae* potently inhibits the ability of antigen-primed bone-marrow-derived DCs (BMDC) to trigger T-cell proliferation by inducing expression of both immunosuppressive cytokines and tolerance-inducing cell surface protein [14]. Furthermore Escobar *et al* [15] recently demonstrated that GC modulates MΦ and their functionality, producing a shift towards anti-inflammatory cytokine production, inefficient upregulation in molecules involved in antigen presentation and T-cell activation and a poor allogeneic T-cell stimulatory activity [15]. These studies showed that *N*. *gonorrhoeae* also suppresses adaptive immune responses through effects on antigen presenting cells (APCs).

Current view of MΦ considers them as a continuum of phenotypes with overlapping expression of cell surface markers, secreted cytokines and chemokines, and transcriptional regulators. In response to various signals, MΦ may undergo classical-M1 (M1-MΦ) or alternative-M2 (M2-MΦ) activation [16]. The M1 phenotype promotes Th1 response and possesses strong microbicidal and tumoricidal activity [17]. In contrast, M2-MФ are involved in parasite clearance, dampen inflammation, promotion of tissue remodeling, tumor progression and possess immune-regulatory functions [16]. M1 and M2 phenotype can be converted into each other in specific microenvironments [18]. During microbial infection, MΦ are polarized to M1 or M2 in response to microbial components and host immune mediators. Depending on the bacterial species, M1 or M2 polarization can play either a beneficial or a detrimental role in disease outcomes [19, 20]. The persistence of bacterial pathogens in tissues and the chronic evolution of infectious diseases are linked to MΦ reprogramming towards heterogeneous M2 signatures. For example, *Coxiella burnetii* elicits an atypical M2 profile in MΦ combining M1/M2 characteristics [19], while *Yersinia enterocolitica* stimulates a clear-cut M2 program in MΦ [21]. The presence of M2 is also critical for the chronic fate of mycobacterial infections, and high levels of M2-derived IL-10 are found in early ulcerative lesions of Buruli disease [22]. Although *N*. *gonorrhoeae* has been reported to modulate MΦ [15, 23], GC influence on MΦ polarization has not been yet explored. In order to address this issue we studied the effect of *N*. *gonorrhoeae* using an *in vitro* model of human monocyte derived MФ (MDM). *N*. *gonorrhoeae* exposure leads to the upregulation of IL-6 and IL-10, which are inflammatory and immunosuppressive cytokines respectively. Interestingly, Programmed Death Ligand 1 (PD-L1) was also induced. However, molecules necessary for an efficient adaptive immune response (CD86, MHCII) were not affected. Consequently we showed that gonococci induce hyporesponsiveness of interacting T cells, demonstrating for the first time that *N*. *gonorrhoeae* interferes with MФ polarization favoring a shift towards a regulatory phenotype.

## Material and Methods

### Blood Samples

Donor buffy coats were obtained to generate macrophages. The study was approved by the local Scientific ethic committee (Hospital Clínico Universidad de Chile, Act approval number 58). All donors provided written informed consent. After the samples had been collected, each donor was allocated a trial number, demographic data were collected and the database anonymised.

### Bacteria and culture conditions

The *Neisseria gonorrhoeae* P9-17 strain used in this study was kindly provided by Dr. Myron Christodoulides (University of Southampton, UK) [24]. In particular, P9-17 (Pil+ Opa_b_+) variant of *N*. *gonorrhoeae* containing the red-shift mutant GFP (rs-GFP) plasmid was used. Bacterial growth and analysis of colony morphology were handled as previously described [15]. Briefly, gonococcal variants were taken from frozen stocks, plated on GC agar plates (Difco, Becton Dickinson) containing BBL Isovitalex (Becton Dickinson, Sparks, MD) and cultured at 37°C in 5% CO_2_ for 18 to 20 hours to obtain single colonies. Single colonies showing the proper morphology were further grown for subsequent experiments.

### Macrophages generation and polarization

Human monocytes were obtained from normal blood donor buffy coats by two-step gradient centrifugation followed by an additional step using the RosetteSep™ Human Monocyte Enrichment Cocktail (STEMCELL Technologies). MΦ were obtained by culturing monocytes (84% CD14+) for 7 days in RPMI 1640 (GIBCO, Invitrogen Corporation) supplemented with 10% FBS (HyClone), 50 U/mL penicillin, 50 μg/mL streptomycin (Gibco Invitrogen) and 50 ng/mL of M-CSF **(**MiltenyiBiotec) in 6-well plates at a density of 2 x 10^6^ cells per well. Polarization was induced by replacing the culture medium for RPMI 1640 supplemented with 5% FBS and 100 ng/mL LPS plus 20 ng/mL IFN-γ (for M1 polarization) or 1000 U/mL IL-4 (for M2 polarization) and culturing cells for an additional 24 hours. Three different cell types were generated: resting fully differentiated 7 days MΦ (M0-MΦ), classically activated (M1-MΦ), and alternatively activated (M2-MΦ).

### Infection of primary macrophages

Gonococcal isolates were taken from frozen stocks and cultured on GC agar plates at 37°C in a 5% CO_2_ atmosphere. Bacteria were then scraped from confluent culture plates and re-suspended in 1 mL of serum-free medium. Bacterial concentration was estimated by optical density at 600 nm (1 O.D unit corresponding to 3.2 x 10^9^ CFU/mL). M0-MΦ were infected with GC at multiplicity of infection (MOI) of 10, 100 or 1000 for 4 hours. Then cultures were supplemented with gentamicin (100 μg/mL) (Invitrogen Corp., Carlsbad, CA) to kill extracellular bacteria. Cultures were returned to 37°C, 5% CO_2_ in humidified incubator and harvested 24 hours post infection for co-culture with T cells or down-stream assays.

### Immunofluorescence microscopy analysis

M0-MΦ were grown on cover slips using antibiotic-free cell culture medium. Nearly confluent cell monolayers were challenged with the rs-GFP GC strain at MOI of 100 and incubated for 4 hours at 37°C with 5% CO_2_. Then cell monolayers were washed five times with medium and fixed for fluorescence microscopy in 1% paraformaldehyde in 1 x PBS (pH 7.4). DAPI and rhodamine-phalloidin staining was carried out for visualizing the nucleus and F-actin respectively. Association of GFP-fluorescent bacteria with stained MФ was determined using epifluorescence microscopy.

### Gentamicin protection assay

Assays were performed as described previously [25]. Briefly, to quantify the total number of MΦ-internalized gonococci, M0-MΦ were infected at MOI 100 for 4 or 8 hours. Next, 100 μg/mL of gentamicin (US Biological, Swampscott, MA) were added in order to kill the extracellular bacteria. Cells were washed 3–5 times with 1 x PBS and lysed with 1% saponin (Sigma, St Louis, MO) in 1 x PBS for 30 min. The lysates were collected, serially diluted and aliquots were seeded onto supplemented GC agar plates and incubated 24 hours at 37°C and 5% CO_2_. Finally, colony forming units (CFU) were counted. To confirm that gentamicin indeed killed all non-internalized bacteria, 50 μL of the infection medium post gentamicin treatment were seeded onto GC plates. No bacterial growth was observed.

### Immunophenotyping and flow cytometry

The following directly conjugated anti-human monoclonal antibodies were used: CD4-FITC, CD4-APC, CD8-PE, CD163-PE, CD206-FITC, CD86-PE-Cy5, CD64-APC, CD273-PE, TLR-4-PE-Cy7, CD40-FITC, HLA-DR-PECy5 and CD274-APC (eBioscience, San Diego). Saturating amounts of antibody were used to stain approximately 3 x 10^5^ cells in staining buffer (1 x PBS, 2% FBS) at a final volume of 20 μl for 30 min at 4°C protected from the light. All samples were washed with staining buffer and resuspended in 200 μl of FACS Buffer. Samples were examined in a FACSCalibur (BD Biosciences) and analysis was performed using FlowJo software (Tree Star, Inc., OR).

### Cytokine detection

IL-10, IL-6, IL-1β, IL-23 and IL-12 levels were measured in supernatants 24 hours post infection or after LPS-IFN-γ/IL-4 treatment (M1/M2 positive control) by enzyme-linked immunosorbent assays using ELISA Ready-SET-Go! (eBioscience, USA), according to the manufacturer’s instructions.

### Mixed lymphocyte reaction (MLR) assay

Peripheral blood lymphocytes (PBL) were obtained from human peripheral blood cells (PBMC) of a single donor. Briefly, PBMC were depleted from antigen-presenting cells by adherence to T75 tissue culture flask supplied with 10% FBS RMPI 1640 medium without agitation. Two hours later, non-adherent (NAD) cells were collected and incubated overnight into another T75 tissue culture. The NAD cells containing PBL were collected and labeled with CFSE (5 mM per 1 × 10^7^ cells) (eBioscience, USA) for 10 min at 37°C. Cells were washed extensively and 2 × 10^5^ cells/well were cultured with 1 × 10^5^ M1, M2 or GC-treated MФ from another donor in round-bottomed 96-well plates in RPMI-1640 medium (Gibco Invitrogen) with 10% fetal bovine serum (FBS, HyClone), 50 U/mL penicillin and 50 μg/mL streptomycin (Gibco Invitrogen) at 37°C, in a 5% CO_2_ atmosphere for 7 days. As a positive control we used PBL stimulated with 150 U/mL of IL-2 and 20 μg/mL of anti-CD3 (OKT-3). Medium was changed at day 3. At day 7, co-cultures were collected and stained against CD4 using the previously described conjugated antibodies. Proliferation analysis was performed using FlowJo software (Tree Star, Inc., OR).

### Quantitative Real Time-PCR (qRT-PCR)

The total RNA was extracted from macrophages as described previously [26]. Reverse transcription of RNA (5 μg) was performed using the Transcriptor First-Strand cDNA synthesis kit following the manufacturer’s recommendations (Roche Applied Science, Mannheim, Germany). To quantify the mRNA expression for the M1 and M2-associated cytokines, 50 ng of cDNA were amplified by quantitative real-time PCR, using the appropriate primers and the Sybr®Green Master Mix (Fermentas) in an ABI PRISM 7900 Sequence Detector System (Applied Biosystems, Foster City, CA, USA). The cycle program used was: 95°C for 10 min, followed by 40 cycles of 95°C for 15 s, 60°C for 30 s, and 72°C for 30 s. The fold change in expression of the target gene relative to the 18S endogenous control was set at 2^-∆∆Ct^, where ∆∆Ct = (Ct_Target_ – Ct_18S_)_stimulated_ – (Ct_Target_ – Ct_18S_)_unstimulated_.

### Statistical analysis

Data was analyzed by two-way ANOVA with Tukey post-test and showed as mean ± standard error (SEM), (Graphpad Prism V5.0). Statistical significance was considered at a p value less than 0.05. The data presented are representative of at least three biological replicates.

## Results

### *Neisseria gonorrhoeae* interaction with macrophages results in a shift towards a M2 macrophage profile

**Fig 1:**
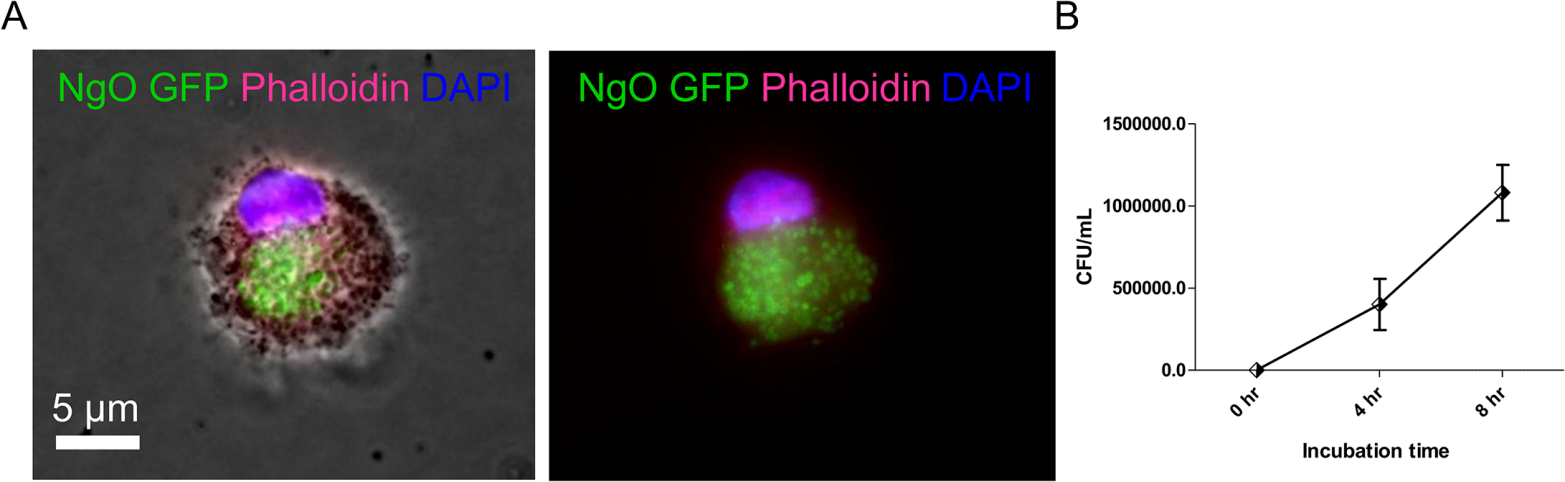
Gonococcus uptake by human MФ. (A) Epifluorescence micrographs of M0-MΦ incubated with rs-GFP GC variant for 4 hours. Right panel shows merge of GFP (green)/ DAPI (blue)/ Rhodamine (red). Left panel is the merge of all three fluorescent channels overlaid on the phase contrast image to denote cell boundaries. (B) Gonococcus internalization by human MФ was evaluated trough a gentamicin protection assay. M0-MΦ were infected at time 0 and after 4 and 8 hours of infection they were treated 1 hour with gentamicin to kill extracellular bacteria. M0-MΦ were then lysed after treatment with saponin for 30 min. Cell lysates were serially diluted, plated in GC agar plates and incubated during 24 hours for CFU counting.

Applying the widely used method to obtain fully differentiated macrophages (M0-MФ) from M-CSF-treated human monocytes [27], we examined whether *N*. *gonorrhoeae* might differentially activate M0-MФ towards a M1 or M2-associated profile. Due to the fact that *N*. *gonorrhoeae* corresponds to facultative intracellular bacteria, we first tested whether M0-MΦ were able to internalize the pathogen. M0-MФ were challenged with the rs-GFP GC variant and then MΦ-internalized bacteria were evaluated by epifluorescence microscopy. Several bacteria were observed in the MΦ cytoplasm 4 hours after infection (Fig 1A). In addition, gonococcus internalization was also evaluated through a gentamicin protection assay, confirming that a significant number of intracellular, viable bacteria could be recovered from the MΦ cytoplasm (Fig 1B). Likewise, the bacterial counts increased at 8 hours post infection given that MΦ were allowed to recognize and internalize bacteria during a longer period of time.

**Fig 2:**
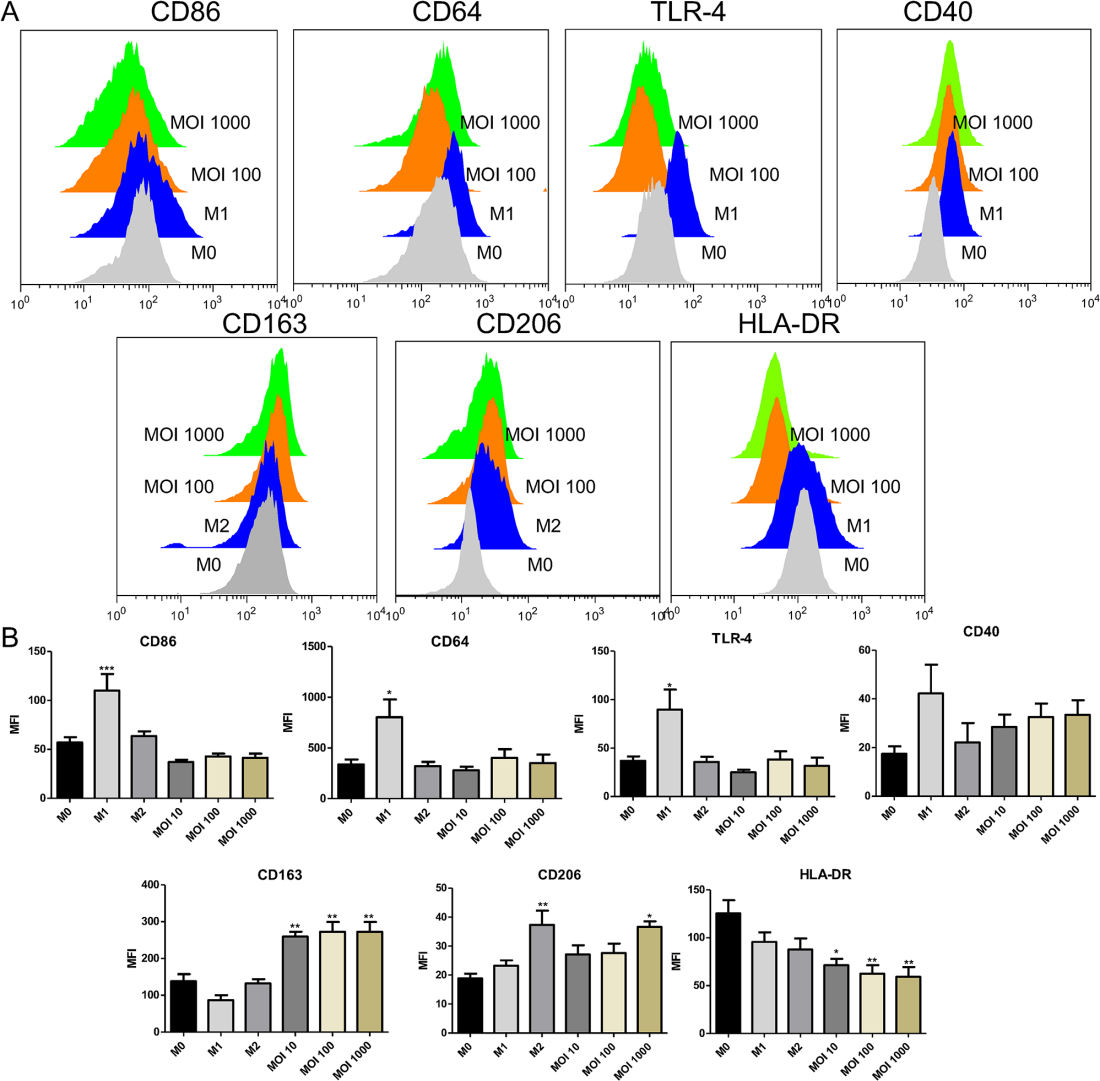
*N. gonorrhoeae* induced M1 and M2-MΦ associated markers. Expression of M1 and M2-MΦ distinctive surface markers were evaluated in GC-treated MΦ by flow cytometry (monocytic cells gated). (A) Representative histograms for each evaluated marker from at least three independent experiments. (B) Mean fluorescence intensity (MFI) average for each marker. Data represent at least 3 independent experiments; bars indicate SEM; * p < 0.05. ** p < 0.01. *** p < 0.001 indicates significant induction compared to non-stimulated MΦ (M0-MΦ).

Once the M0-MΦ capacity to interact and internalize *N*. *gonorrhoeae* is confirmedwe evaluated several M1 and M2-MФ-associated markers by flow cytometry 24 hours post *N*. *gonorrhoeae* exposure. Stimulation with *N*. *gonorrhoeae* increased the expression of the M2-MФ-associated marker CD163 at all the MOIs tested. CD206 M2-MФ-associated marker, in contrast, was only increased at MOI 1000 (Fig 2).

Regarding M1-MФ-associated markers, CD64 and TLR-4 were significantly upregulated upon treatment with LPS/IFN-γ (M1-MΦ) as expected. Yet these markers were not induced in GC-treated MΦ, which exhibited similar levels to the non-stimulated M0-MΦ. Moreover *N*. *gonorrhoeae* was not able to induce the co-stimulatory CD86 neither the major histocompatibility complex (MHC class II) molecules. Finally CD40, another M1-MΦ–associated marker, showed a tendency towards upregulation upon bacteria treatment, although no statistically significant differences were observed between the different treatments.

### *N*. *gonorrhoeae* induces a mixed cytokine profile in human macrophages

**Fig 3:**
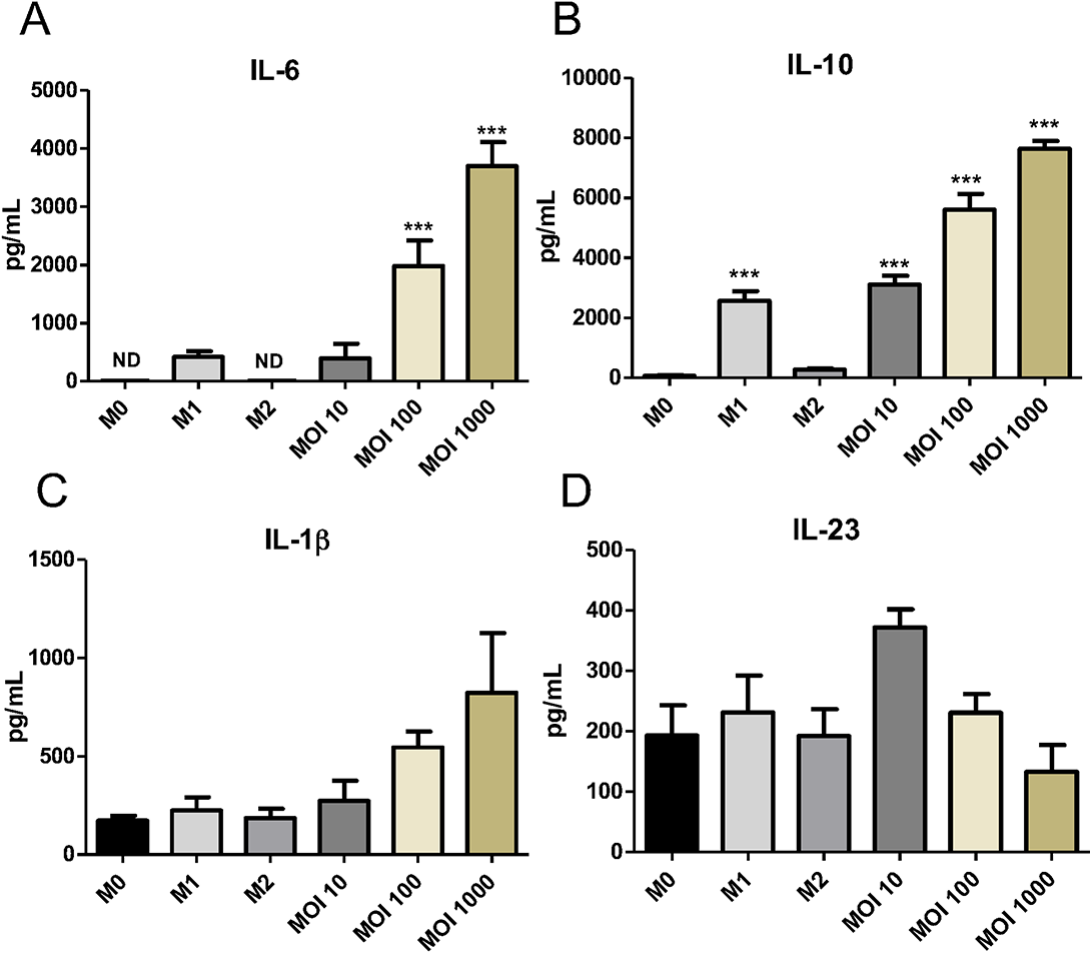
*N. gonorrhoeae* induced a mixed cytokine profile in human MФ. (A) IL-6, (B) IL-10, (C) IL-1β and (D) IL-23 cytokines were evaluated 24 hours post infection with *N*. *gonorrhoeae* or M1/M2 polarization. Data obtained are expressed as the mean ± SEM and represent at least three independent experiments. ** p < 0.01. *** p < 0.001 indicates significant induction compared to non-stimulated MΦ (M0-MΦ). ND. non detected.

Considering that microorganisms can modulate the MФ phenotype [28], we aimed to determine the effects of gonococcus on MΦ functionality by evaluating the cytokine profile induced by *N*. *gonorrhoeae*. The pro-inflammatory M1-MФ-associated cytokines IL-6, IL-1β, and IL-23, as well as the anti-inflammatory cytokine IL-10-characteristic of the M2-MФ subtype-, were measured in culture supernatants (Fig 3A–3D). Data obtained from 6 independent experiments 24 hours after challenge revealed that GC at MOI of 100 and 1000 significantly induced the production of the pro-inflammatory cytokine IL-6 in comparison to M0-MФ (Fig 3A). A similar result was observed for IL-10 (Fig 3B) but at a lower dose of *N*. *gonorrhoeae* (MOI 10). In other words, infected macrophages produced IL-10 rather than IL-6 and IL-1β. Even though IL-1β and IL-23 levels did not reach statistically significant differences between GC-treated MΦ and M0-MΦ, these cytokines exhibited a tendency to increase and decrease in a dose dependent manner respectively (Fig 3C and 3D). Additionally, qRT-PCR analysis 4 hours post infection confirmed the results described above in the sense that GC-infected macrophages upregulated mRNA expression of IL-10 and IL-6. Remarkably TNF-α mRNA was also induced (S1 Fig). It is important to mention that even though TNF-α and IL-6 are M1-MΦ-associated cytokines, they are also characteristic of the M2b-MΦ subtype which also produces IL-10 [28]. Therefore, our results suggest that *N*. *gonorrhoeae* polarize human MΦ towards a M2 profile, particularly a M2b-MΦ subtype.

### *N*. *gonorrhoeae*-exposed macrophages upregulate the co-inhibitory molecule PD-L1

**Fig 4:**
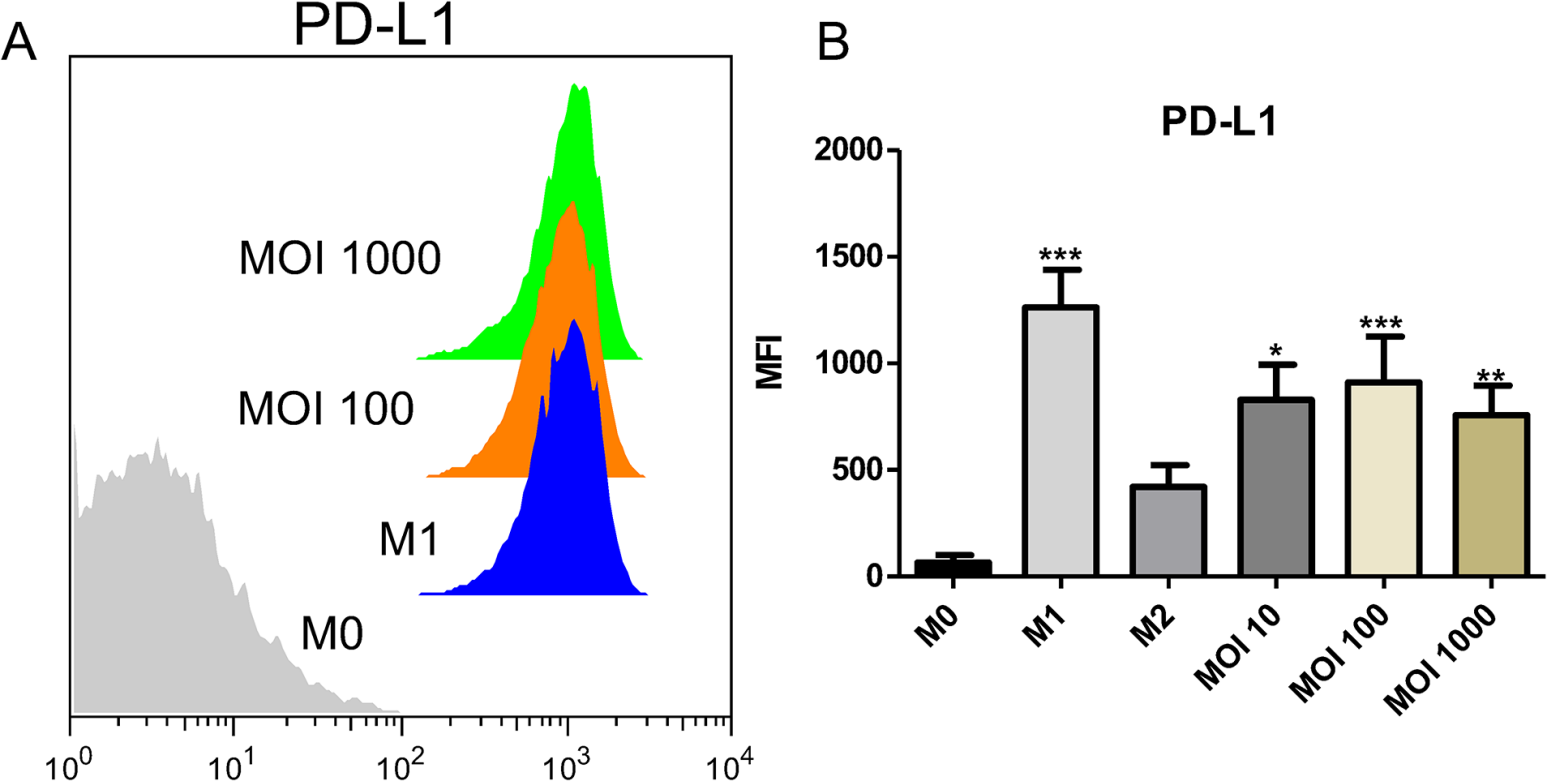
PD-L1 expression was upregulated in human MФ upon *N*. *gonorrhoeae* infection. Human MФ treated for 24 hours with medium only, or M1/M2 polarizing stimulus, or *N*. *gonorrhoeae* (MOI = 10, 100, 1000) were immunostained for flow cytometric analysis of PD-L1. (A) Representative overlay histograms. (B) MFI average. Data obtained are expressed as the mean ± SEM and represent at least nine independent experiments. * p < 0.05. ** p < 0.01. *** p < 0.001 indicates significant induction compared to non-stimulated MΦ (M0-MΦ).

Other molecule we thought interesting to study in the macrophage-GC context was PD-L1. PD-L1 is a member of the co-stimulatory family of proteins and it is involved in the regulation of the immune response [29–32]. Several reports indicate that PD-L1 participates in the generation of Tregs and in maintaining self-tolerance [33–35] According to our flow cytometry results, we found a significant upregulation of PD-L1 in M0-MΦ upon gonococcal infection (Fig 4). These data along with secreted IL-10 levels suggest that *N*. *gonorrhoeae* polarize M0-MΦ towards a more likely regulatory macrophage.

### Allostimulatory capacity is deficient on *N*. *gonorrhoeae*-infected MΦ

**Fig 5:**
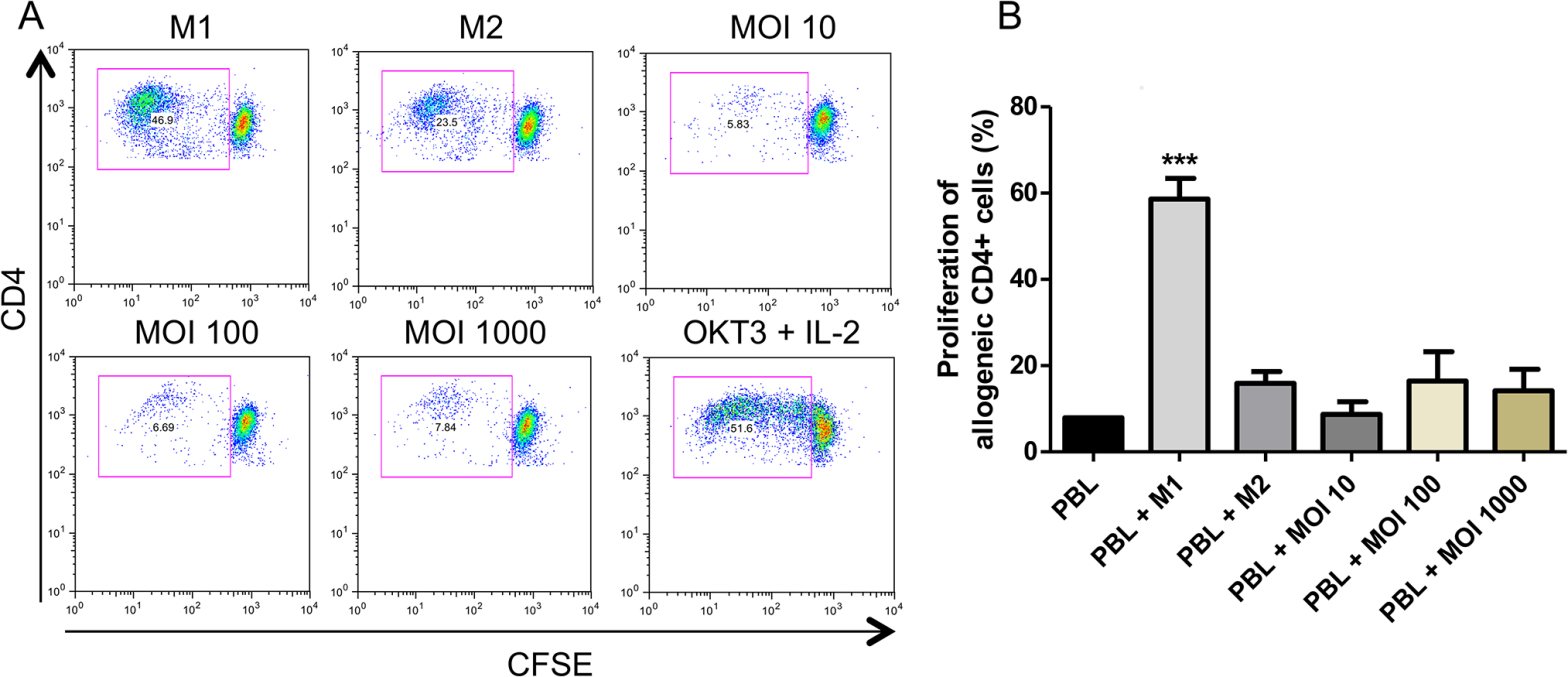
Hyporesponsive alloantigen T-cell responses induced by MФ infected with *N*. *gonorrhoeae*. CFSE-labeled CD4+ cells proliferation after non-adherent cells were co-cultured with human MФ treated for 24 hours with LPS-IFN-γ (M1-MΦ), IL-4 (M2-MΦ) or *N*. *gonorrhoeae* (MOI = 10, 100, 1000) for 7 days at the ratio of 2:1. As a positive control of proliferation we used PBL stimulated with 150 U/mL of IL-2 and 20 μg/mL of anti-CD3 (OKT-3). (A) Representative T CD4+ cell proliferation dot plots from one of the donors are shown. (B) CD4 + cells proliferation average under different conditions. ** p < 0.01 indicates that only M1-MФ profile is able to significantly induce proliferation of CD4+ cells in a mixed lymphocyte reaction.

Since the surface markers and cytokines profile induced by *N*. *gonorrhoeae* on infected MΦ are well-matched with M2 profile, we addressed to study the capacity of GC-treated MФ to stimulate T cells. PBL from a single donor were co-cultured with GC-treated MФ (or M1/M2-MФ for the controls) from another donor in a mixed lymphocyte reaction (MLR) assay. After 7 days of co-culture with *N*. *gonorrhoeae*-treated MФ, CD4+ cells exhibited no significant proliferation, evaluated trough CFSE dilution, as compared to M1-MΦ-exposed cells (Fig 5). Although T cell proliferation was evaluated at three different MOIs (10, 100 and 1000), we did not observe significant differences between them.

## Discussion

It has been previously demonstrated that *N*. *gonorrhoeae* is capable of inducing a tolerogenic profile not only in RAW murine macrophage cell line but also in human dendritic cells [14, 15]. However, the effect of gonococcal infection in human MФ has not been yet reported. In this study we first evaluated the effect of *N*. *gonorrhoeae* at different doses in the polarization of MDM (referred to as M0-MФ). M1-MФ (or classically activated) and M2-MФ (or alternatively activated) associated surface markers were measured by flow cytometry. We found *N*. *gonorrhoeae* was indeed capable of inducing CD163 in M0-MФ at the lowest bacterial concentration (MOI 10) whereas CD206 was only induced at MOI 1000. M2-MФ profile includes at least three subsets: M2a, induced by IL-4 or IL-13; M2b, induced by immune complexes and agonists of TLRs or IL-1 receptors; and M2c, induced by IL-10 and glucocorticoid hormones [36]. Although CD206 is the best characterized M2-MФ marker, as is present in all the M2-MФ subtypes, there is controversy regarding CD163 expression on M2a phenotype (referred to as M2-MФ in our study). Specifically, Zizzo *et al* [37] state that M1 and M2a-MФ (generated upon stimulation with IL-4) exhibit low levels of CD163. Vogel *et al* [38] also determined CD163 did not differ significantly in M2a-MФ compared to M0-MФ. This might explain why we did not observe an upregulation of CD163 in our M2-MФ control.

Unlike M0-MФ treated with LPS/IFN-γ, *N*. *gonorrhoeae* was not able to induce the M1-MФ-associated markers CD86, MHCII, TLR-4 nor CD64 at any of the three bacterial doses tested (Fig 2). Indeed, the expression levels exhibited after GC-stimulation were similar to those observed in M2 and M0-MФ controls. Although CD40 showed a tendency towards upregulation upon infection, no significant differences were observed in comparison with the M0-MФ control, neither between M0-MФ and the M1-MФ positive control. The latter suggests that CD40 is not a suitable marker of the M1-MФ phenotype, which differs from other studies that have established CD40 as the most distinctive M1-MФ profile marker [38]. Interestingly, the lack of induction of the cell surface co-stimulatory molecule CD86 upon infection with *N*. *gonorrhoeae* seems to be an infrequent feature of pathogens in many studies using transcriptional tools, which have indicated that CD86 along with other M1-MФ markers, −including cytokines such as TNF, IL-6, IL-1β− are upregulated upon infection with several bacteria for instance, *Yersinia enterocolitica*, *Tropheryma whipplei* [28], *Salmonella enterica* serovar Typhimurium [39] and *Mycobacterium tuberculosis* [40]. Since the CD86 and the MHC-II molecules are extremely necessary to antigen presentation, it is likely that the MФ resulting from the infection with *N*. *gonorrhoeae* have a poor proliferative capacity over T cells. These data are supported by a previous report where *N*. *gonorrhoeae* was unable to induce significant upregulation of neither CD86 nor MHC class II in the murine MФ cell line RAW [15]. Although *N*. *gonorrhoeae* is actually phagocytosed by MФ (Fig 1), our data suggest that the bacteria might weaken antigen-presenting functions because the immune responses regulated by the CD86/CD28 co-stimulatory pathway are impaired in the absence of CD28 signaling. As is known, these immune responses are responsible of antibody production and induction of cytotoxic T-cell activity [41]. In addition, the low levels of TLR-4 exhibited by GC-infected MФ might lead to deficient activation of APCs, thus resulting in chronic infection with weakened bacterium elimination as previously reported in a mycobacterial model using TLR-4 mutant mice [42]. CD64, also known as FcγR1, is another well-characterized M1-MФ-associate marker that was not induced upon infection with *N*. *gonorrhoeae* (Fig 2). CD64 belongs to the Fcγ family of receptors and binds IgG with high affinity [43]. Importantly, upon Fc binding, the CD64 receptor induces the association of the γ chain, triggering functional responses such as phagocytosis. Binding of CD64 with IgG also mediates antibody-dependent cellular cytotoxicity (ADCC) as well as induction of several cytokine genes transcription and release of inflammatory mediators [44]. Based on the above, we suggest that the low expression of this receptor on MФ infected with *N*. *gonorrhoeae* might help gonococcus to evade some immune responses, especially the ADCC-mediated response.

Once established the surface marker profile exhibited by GC-treated MФ, we evaluated their functional polarization through the release of M1-MФ and M2-MФ-associated cytokines. Interestingly, infection with *N*. *gonorrhoeae* significantly induced pro (IL-6 and TNF-α) and anti-inflammatory (IL-10) cytokines in M0-MФ (Fig 3 and S1 Fig). IL-6 secretion triggered by *N*. *gonorrhoeae* infection has been observed *in vivo* [45]. In particular, it has been demonstrated that GC not only induces the secretion of the pro-inflammatory cytokine IL-6 but also TNF-α in APCs that are located in the stroma of the female mouse genital tract. This is supported by the increased levels of TNF-α and IL-6 observed *in vivo* in vaginal secretions of Balb/c mice after gonococcal infection [45]. *In vitro*, Feinen *et al* [5] further demonstrated that BMDC cultured with *N*. *gonorrhoeae* also produced IL-6 along with IL-23, but not IL-12. Moreover, upon stimulation with *N*. *gonorrhoeae*, human THP-1-derived MФ also secreted IL-6 and IL-23, 1β and TNF-α, but not IL-12, which suggest that human and mouse APCs behave similarly in response to GC-stimulation. In our model, although IL-1β and IL-23 were also induced upon infection, we did not obtain statistically significant differences between the unstimulated/stimulated MФ. These data suggest that although response to *N*. *gonorrhoeae* might trigger some inflammatory pathways (IL-6 production); this is not sufficient to activate the adaptive immune system through co-stimulatory molecule induction. This would possibly result in a chronic inflammatory condition without clearance of the pathogen as observed in infected patients [7]. Remarkably we did not detect IL-12 secretion in *N*. *gonorrhoeae*-infected MФ neither in the positive control M1-MФ (data not shown), which is in accordance with previous studies [5, 45]. An explanation for this is M1-MФ also released significant levels of IL-10, which in turns might inhibit IL-12 secretion.

Interestingly, IL-10 was strongly induced in a dose-dependent manner upon infection with *N*. *gonorrhoeae*. IL-10 induction by *N*. *gonorrhoeae* was recently demonstrated by a study of Liu *et al* [10] which showed both *in vitro* and *in vivo* that *N*. *gonorrhoeae* strongly induced IL-10 and Tr1 cells.

IL-10 is one of the most important regulatory cytokines and it is induced following stimulation with TLR ligands such as LPS [46]. This fact explains the low levels of IL-10 secreted by control M2-MФ (Fig 3). The role of IL-10 has also been observed upon infection with other pathogens. Particularly, in lepromatous lesions caused by *Mycobacterium ulcerans*, in infections with *Coxiella burnetii* and *Mycobacterium tuberculosis* [19, 22].

The cytokine profile (IL-10, IL-6, TNF-α) elicited upon infection with *N*. *gonorrhoeae* correlates well with the M2b-MФ phenotype. Furthermore M2b-MФ as well as GC-infected macrophages exhibit CD163 marker on their surface [28]. Our findings lead us to suggest *N*. *gonorrhoeae* stimulates a M2-MФ phenotype in which some of the M2b and none of the M1-MФ-associated markers are induced.

Besides the assessment of IL-10 production, we evaluated other surface markers with immunosuppressive properties, in particular Programmed Death Ligand 1 (PD-L1), on infected MФ. PD-L1 is important in suppressing the immune system during specific events such as autoimmune diseases and pregnancy [47–50]. In this work, we found PD-L1 was significantly induced upon *N*. *gonorrhoeae* exposure in all the MOIs tested (Fig 4). It is important to mention that PD-L1 was also upregulated in M1-MФ control. However, upregulation of CD86 expression in M1-MФ was also observed (Fig 2B). Furthermore, the effect of *N*. *gonorrhoeae* in PD-L1 has been documented by Zhu *et al* [14]. Particularly, they observed PD-L1 upregulation upon *N*. *gonorrhoeae* exposure in primary human DCs and murine bone marrow derived DCs (BMDCs). Although little is known about the role of PD-L1 in MФ during bacterial infections, several studies have reported this role in DCs [44, 51, 52]. Specifically, PD-L1-mediated DC immunosuppression has been observed in response to commensally or pathogenic bacteria that colonize the genital tract [53, 54].

In order to determine the functionality of *N*. *gonorrhoeae*-stimulated MФ and to evaluate whether it might confer them a regulatory phenotype, *N*. *gonorrhoeae*-treated MФ were co-cultured with allogenic CFSE labeled PBL. It was found that these MФ were not able to induce CD4+ T cell proliferation, unlike our positive control M1-MФ did (Fig 5). These data correlate with our previous study in which it was demonstrated that GC-treated RAW cells possess weak allogeneic T-cell stimulatory activity [14, 15]. In addition, Zhu *et al* [14] showed that GC-exposed BMDCs and also human DCs failed to elicit antigen-induced CD4+ T lymphocyte proliferation. Although further studies are needed to determine which is the exactly mechanisms responsible of the hyporesponsive alloantigen responses exhibited by T cells upon stimulation with *N*. *gonorrhoeae*-infected MФ, our work reports a novel strategy by which *N*. *gonorrhoeae* modulates host innate immune response by polarizing M0-MΦ towards a regulatory/M2-MΦ phenotype and provides new insights that might help to unravel the complexity of the immune response against gonococcal infection.

## Supporting Information

S1 Fig Transcriptional cytokine analysis of *N*. *gonorrhoeae*-stimulated human MФ. Quantitative PCR analysis for cytokine mRNA expression on *N*. *gonorrhoeae*-stimulated MΦ. M1 and M2-MΦ were used as controls. Log_2_ expression levels for IL-10, IL-6, IL-1β, TNF-α and IL-23. Results are expressed as the ratio of the expression level in stimulated vs. unstimulated MΦ (M0-MΦ) and represent the mean ± SEM of three independent experiments.
(TIF)
