# Supplementary figures and images for: Neisseria gonorrhoeae Modulates Immunity by Polarizing Human Macrophages to a M2 Profile
Source: PLoS One. 2015 Jun 30;10(6):e0130713. doi: 10.1371/journal.pone.0130713 (PMC4488386; doi:10.1371/journal.pone.0130713)

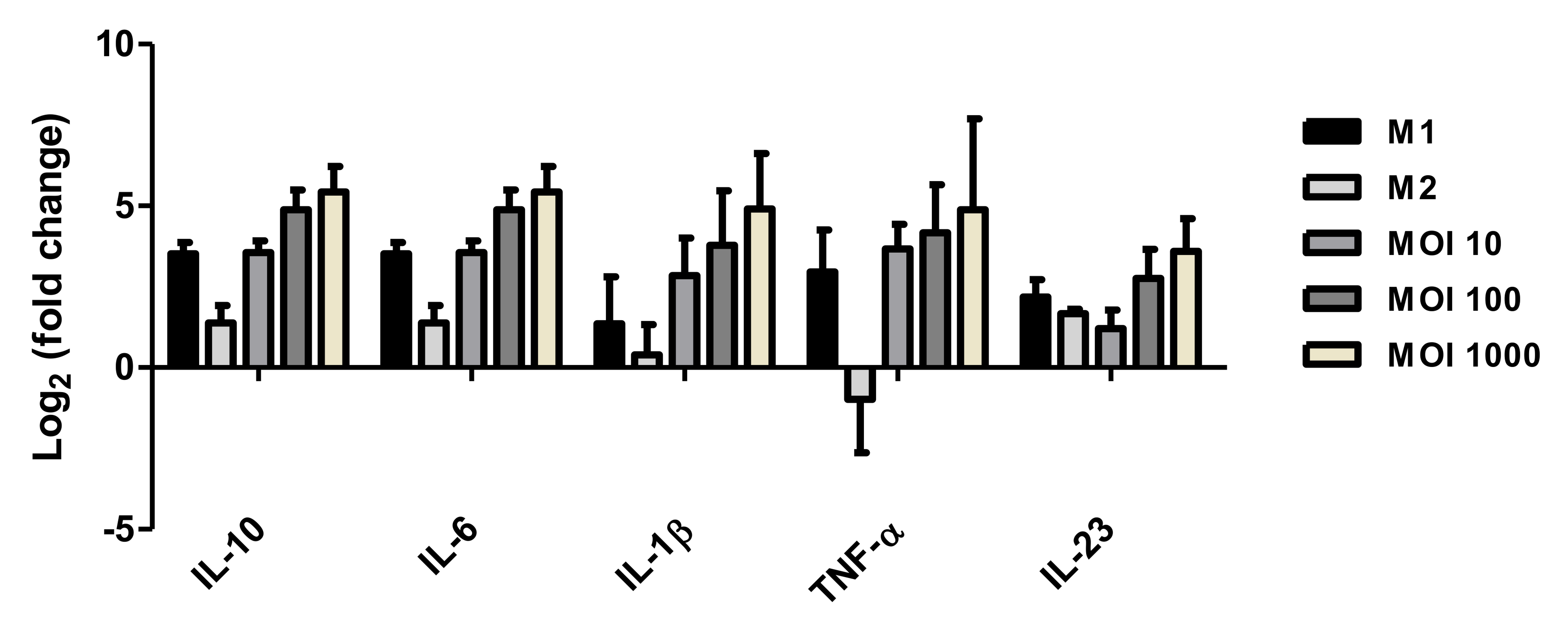

Supplement: S1 Fig — Quantitative PCR analysis for cytokine mRNA expression on N. gonorrhoeae-stimulated MΦ. M1 and M2-MΦ were used as controls. Log2 expression levels for IL-10, IL-6, IL-1β, TNF-α and IL-23. Results are expressed as the ratio of the expression level in stimulated vs. unstimulated MΦ (M0-MΦ) and represent the mean ± SEM of three independent experiments. (TIF) [file pone.0130713.s001.tif]
